# Acute Hypercapnic Respiratory Failure from Foreign Body Aspiration in a 16-Month-Old: A Case Report

**DOI:** 10.5811/cpcem.47015

**Published:** 2025-12-07

**Authors:** Sabrina Lee, Richard D. Shin, Kallie Combs

**Affiliations:** New York-Presbyterian Queens, Department of Emergency Medicine, Flushing, New York

**Keywords:** foreign body aspiration, acute hypercapnic respiratory failure, pediatric resuscitation, case report

## Abstract

**Introduction:**

Acute hypercapnic respiratory failure secondary to foreign body aspiration is a rare but severe complication seen in pediatric patients. Foreign body aspiration is one of the leading causes of death in children and requires prompt intervention and stabilization when definitive bronchoscopy is not readily available.

**Case Report:**

We describe the case of a 16-month-old male who developed acute hypercapnic respiratory failure following the aspiration of a foreign body. On presentation to the emergency department, the child was in respiratory distress, appeared cyanotic, and had severely impaired oxygenation, all indicating respiratory failure. Initial management involved stabilization, advanced airway management, and ventilatory adjustments. Efficient communication with multiple specialists coordinated the appropriate transfer of the patient to a tertiary pediatric facility for bronchoscopy and ultimate successful foreign body removal without complications.

**Conclusion:**

The report highlights challenges in the management of pediatric foreign body aspiration leading to severe hypercapnia, the importance of interdisciplinary coordination, and the management techniques used to stabilize the patient for safe transfer to a tertiary care center.

## INTRODUCTION

Foreign body aspiration is a common cause of respiratory compromise in children and one of the leading causes of accidental deaths among children with morbidity ranging from 10% to 20% worldwide.^1^ In the United States, foreign body aspiration is the leading cause of accidental deaths in children under six years of age.^1^ The main symptoms of foreign body aspiration include acute onset of severe coughing, wheezing, dyspnea, or stridor. Often, as the foreign body ceases movement in the respiratory tract, these acute symptoms may subside or disappear, allowing this acute episode to bypass the adults’ attention.^2^ Additionally, the absence of these symptoms do not rule out foreign body aspiration when presented with the appropriate clinical presentation. A history of acute “choking” is a critical indicator of respiratory distress and, thus, there should be high suspicion for foreign body aspiration.

Chest radiograph is often the main initial tool for airway foreign body assessment. Common findings include atelectasis, air trapping, pneumothorax, and emphysema. However, one retrospective study found that 32.2% of cases were associated with normal chest radiographs.^3^ Similarly, most foreign bodies that children aspirate on, such as toys, food, and balloons, are radiolucent on chest radiographs.^4^ The gold standard for detection and treatment is rigid bronchoscopy. With high suspicion of foreign body aspiration, bronchoscopy should be used despite the absence of symptoms, physical exam findings, or radiographic findings.

Acute hypercapnic respiratory failure is a rare but potentially life-threatening complication of foreign body aspiration in the pediatric population. When a patient aspirates on a foreign body, there is varying obstruction to the upper and lower airways, impeding adequate ventilation and oxygenation. Poor ventilation leads to the inability to appropriately eliminate carbon dioxide (CO_2_) from the body, contributing to elevated levels of CO_2_ in the blood. Infants and younger children are more likely to experience acute respiratory failure due to a multitude of factors, including decreased respiratory drive in the early days of life; increased airway resistance secondary to reduced nostril and airway diameter; greater collapsibility of respiratory musculature; and lower threshold for respiratory fatigue.^5^ While the typical management involves bronchoscopy for foreign body removal, severe respiratory compromise leading to acute hypercapnic respiratory failure calls for immediate critical intervention.

The literature is limited regarding foreign body aspiration complicated by hypercapnic respiratory failure among pediatric patients. This case illustrates a unique presentation and management of foreign body aspiration in a boy who presented with acute hypercapnic respiratory failure, which required rapid diagnosis, early intervention, and appropriate transfer for specialized care. The patient was successfully treated with removal of the foreign body and had an uncomplicated recovery.

## CASE REPORT

A 16-month-old male with no significant medical history presented to the emergency department (ED) following a witnessed accidental aspiration of a soft toy in the shape of a banana. According to his mother, the child turned purple and exhibited signs of choking shortly after the incident. The parents were unable to dislodge the object with back slaps and brought him to the ED within 10 minutes of the incident. On initial assessment, the child was cyanotic with severely compromised oxygen levels, showing a peripheral oxygen saturation (SpO_2_) of 68% on room air, as well as tachypnea with a respiratory rate elevated to 60 breaths per minute. The child appeared limp and obtunded, although he did have spontaneous eye-opening. Physical examination revealed facial petechiae, which is a clinical sign of significant respiratory effort, and diminished breath sounds on the right side.

*CPC-EM Capsule*

What do we already know about this clinical entity?

*Respiratory failure secondary to foreign body aspiration in children is a life-threatening emergency, which requires prompt recognition and intervention.*

What makes this presentation of disease reportable?

*This case highlights the challenges in managing a difficult pediatric airway, involving patient stabilization, multi-disciplinary consultations, and transfer to a tertiary facility.*

What is the major learning point?

*Early diagnosis and airway management in pediatric foreign body aspiration is essential to ensure timely bronchoscopy and prevent life-threatening outcomes.*

How might this improve emergency medicine practice?

*We review the methods to navigate hypercapnia in pediatric respiratory failure that can improve survival and reduce morbidity.*

The patient was quickly placed on a non-rebreather mask with a flow rate of 15 liters per minute, leading to an immediate improvement in his oxygen saturation to 100%. A chest radiograph was performed, which confirmed the presence of the foreign body lodged in the carina (Image). Initial arterial blood gas showed acidosis secondary to hypercapnia with a pH of 7.07 and partial pressure of arterial CO_2_ (PaCO_2_) of 72 millimeters of mercury (mm Hg) (Table 1). Due to his severe respiratory distress and hypoxia, the child required urgent intubation. Rapid sequence intubation with intravenous midazolam and rocuronium, using video laryngoscopy and a 3.5-mm cuffed endotracheal tube, was successful. During intubation, the foreign body was not visualized as it was near the carina, and moderate airway edema was observed, underscoring the difficulty of maintaining adequate ventilation.

Ventilatory management began with efforts to improve the patient’s oxygenation and reduce his hypercapnia. Initial ventilation attempts revealed hypoxia with SpO_2_ of about 80% and critically elevated end-tidal CO_2_ (ETCO_2_) levels remaining above 100 mm Hg (reference range: 35–45 mm Hg). Repeat arterial blood gas showed worsening of acidosis with a pH of 6.89 and severe hypercapnia of PaCO_2_ above 150 mm Hg (35–50 mm Hg) (Table 2). On reassessment, the patient had diffuse bilateral wheezing, and he was given IV dexamethasone and nebulized albuterol. To address breath stacking, which further compromised his oxygenation, the patient was disconnected from the ventilator periodically, and manual sustained pressure was applied to the sternum. This manual decompression of the chest aided in temporary improvement in oxygenation.

When reconnected to the ventilator, volume control setting was employed at 4–6 milliliters per kilogram (mL/kg) tidal volumes, and pressure limits were set to manage airway pressures; however, peak pressure remained elevated to 55 cm of water pressure (cm H_2_O) (reference range: 30–35 cm H_2_O). Given the concern for risk of barotrauma, ventilator settings were then changed to pressure control with a maximum pressure of 40 cm H_2_O. Even on these settings, the ventilator returned only 5–10 mL of tidal volume, which was significantly suboptimal in a 10.3 kg child. However, this setting showed appropriate rise of SpO_2_ to about 90% and lowering of ETCO_2_ to about 40 mm Hg, indicating improved oxygenation and ventilation; therefore, the patient was ultimately monitored on pressure control settings.

Due to ongoing agitation and poor ventilator synchrony, we administered continuous sedation with IV midazolam, pain control with fentanyl infusion, and paralysis with vecuronium infusion to maintain ventilator control. The patient’s need for rigid bronchoscopy necessitated consultation with pediatric otolaryngologist for foreign body removal. However, given the lack of pediatric bronchoscopy tools in-house, a transfer to a tertiary pediatric facility was organized. Additional planning included obtaining a pediatric chest tube kit from the neonatal intensive care unit, as the risk of pneumothorax was high due to elevated airway pressures. Following transfer to a specialized pediatric center, the patient underwent a successful rigid bronchoscopy, with removal of the foreign body from his airway. He was extubated post-procedure and continued to improve, ultimately being discharged with no further complications.

## DISCUSSION

With foreign body aspiration being an unfortunate, common occurrence within the pediatric population, this case highlights the multiple steps involved in managing the clinical complexities of acute hypercapnic respiratory failure in pediatric foreign body aspiration. Firstly, early recognition is critical to prevent the progression to respiratory failure and allow for earlier targeted management. Foreign body aspiration is a preventable cause of death in children < 3 years of age, an age group that is prone to unwitnessed aspiration due to mouthing behaviors.^6^,^7^ Foreign bodies typically lodge in the bronchi and lower airway, although laryngotracheal obstructions can mimic upper airway conditions such as croup and asthma.^8^,^9^

In a retrospective study, Molla et al recommended maintaining a high index of suspicion for foreign body aspiration in cases of bronchopulmonary infections with an atypical course.^6^ Our patient had a witnessed aspiration, but in cases of unwitnessed events or sudden respiratory symptoms, clinicians should strongly consider foreign body aspiration.^2^,^3^ Sandhofer et al described a child treated for acute bronchitis with worsening symptoms and was found to have a concomitant peanut aspiration.^10^ Pradhan et al also identified a toy in the bronchus of a child evaluated for pnuemonia.^11^ These cases emphasize the importance of thorough history-taking in suspected aspiration cases.

Our patient had severe respiratory distress with hypoxia, prompting intubation with video laryngoscopy. Once on mechanical ventilation, standard ventilator settings resulted in high pressures and suboptimal oxygenation; the patient was then disconnected from the ventilator, and manual chest decompressions offered temporary relief. A similar technique has been used in children with severe asthma and bronchiolitis in the pediatric intensive care unit with significant air trapping. Intermittent manual external chest compression led to gradual but effective improvement in the children’s partial pressure of CO_2_, preventing intubation and allowing for ultimate discharge from the unit.^12^ With external manual decompression, optimal sedation was crucial for ventilator synchrony and oxygenation. Through these maneuvers, the patient was sufficiently stabilized for immediate transfer pending definitive treatment.

The child’s ability to tolerate extreme hypercapnia demonstrates that pediatric patients may withstand elevated CO_2_ levels if oxygenation and circulation are adequately maintained. Following successful removal of the foreign body, the patient was extubated, improved clinically the next morning, and was ultimately discharged two days later without complications. Similar outcomes were seen in a case involving massive grain aspiration with severe hypercapnia (PaCO_2_ of 501 mm Hg), where preserved cardiovascular function allowed for the child’s full recovery.^13^ Likewise, Mazzeo et al reported a boy with sustained hypercapnia with a PaCO_2_ of 293 mm Hg for 14 hours during a near-fatal asthma attack, who remained stable secondary to adequate perfusion and oxygenation.^14^ These cases highlight the resilience of pediatric patients with extensive hypercapnia when oxygenation, perfusion, and cardiovascular function are preserved.

Finally, the need for coordinated, interdisciplinary care was underscored in this case, as successful management required the involvement of pediatric otolaryngology, intensive care, and ED teams. Timely communication between consultants and readiness for potential complications was critical in stabilizing the patient for transfer. The case further illustrates the importance of anticipating potential complications, such as pneumothorax, and preparing proactively to ensure patient safety during transport. With these modalities, our patient was promptly transferred to the tertiary pediatric facility and underwent rigid bronchoscopy with successful removal of the foreign body within two hours of the initial event. This case emphasizes the importance of both prompt intervention and recognition of foreign body aspiration in toddlers, particularly when severe respiratory distress and hypercapnia are present. Through ventilatory support adjustments, consultant coordination, and anticipation of potential complications, the patient was successfully stabilized for treatment and recovery.

## CONCLUSION

In managing pediatric foreign body aspiration leading to severe respiratory distress, several key learning points emerged. First, addressing breath-stacking by disconnecting the patient from the ventilator and manually decompressing the chest can improve ventilation temporarily. Second, in addition to adjusting ventilator control settings, full paralysis can help achieve effective ventilator control in cases with severe respiratory distress. Third, in cases requiring advanced airway procedures, ensuring all necessary consultants are prepared with equipment for safe transfer is essential. Finally, clear communication and collaboration across disciplines can expedite care and improve patient outcomes in critical pediatric emergencies.

## Figures and Tables

**Image:**
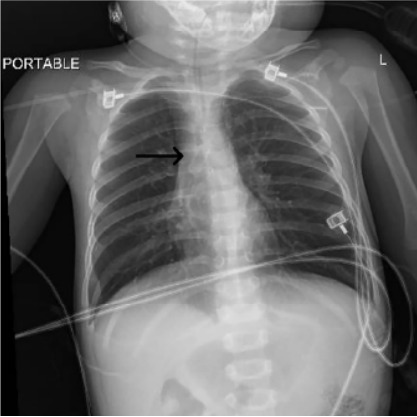
Chest radiograph of a 16-month male with foreign body (arrow) after endotracheal intubation.

**Table 1:** Initial arterial blood gas of a 16-month male upon arrival to the emergency department.

Test Name	Patient Value	Reference Range
pH (Arterial)	7.07	7.32–7.45
pCO_2_ (Arterial)	72	35–50 mm Hg
pO_2_ (Arterial)	363	60–90 mm Hg
HCO_3_ (Arterial)	21.0	17.0–23.0 mmol/L
CO_2_, Total Arterial	22	17–24 mmol/L
Base Excess (Arterial)	−9.90	−6.00–2.00 mmol/L
O_2_ Saturation (Arterial, Calc)	100	95–98%
Glucose WB	319	50–80 mg/dL
Lactate W/B (A)	2.42	mmol/L

*CO_2_*, carbon dioxide; *HCO_3_*, bicarbonate; *O_2_*, oxygen*; pCO_2_*, partial pressure of carbon dioxide; *pO_2_*, partial pressure of oxygen; *mm Hg*, millimeters of mercury; *mmol/L*, millimole per liter; *mmol/dL*, millimole per deciliter; *WB*, whole blood; *W/B (A)*, whole blood arterial.

**Table 2:** Arterial blood gas results of a 16-month male after intubation.

Test Name	Patient Value	Reference Range
pH (Arterial)	6.89	7.32–7.45
pCO_2_ (Arterial)	>150	35–50 mm Hg
pO_2_ (Arterial)	571	60–90 mm Hg
HCO_3_ (Arterial)	cnc	17.0–23.0 mmol/L
CO_2_, Total Arterial	cnc	17–24 mmol/L
Base Excess (Arterial)	cnc	−6.00–2.00 mmol/L
O_2_ Saturation (Arterial, Calc)	cnc	95–98%
Glucose WB	312	50–80 mg/dL
Lactate W/B (A)	< 0.30	mmol/L

*CO_2_*, carbon dioxide; *cnc*, could not calculate*; HCO_3_*, bicarbonate; *mm Hg*, millimeters of mercury; *O_2_*, oxygen*; pCO_2_*, partial pressure of carbon dioxide; *pO_2_*, partial pressure of oxygen; *mm Hg*, millimeters of mercury; *mmol/L*, millimole per liter; *mmol/dL*, millimole per deciliter; *WB*, whole blood; *W/B (A)*, whole blood arterial.
